# Efficacy of cell-free DNA methylation-based blood test for colorectal cancer screening in high-risk population: a prospective cohort study

**DOI:** 10.1186/s12943-023-01866-z

**Published:** 2023-09-28

**Authors:** Fuqiang Zhao, Ping Bai, Jianfeng Xu, Zitong Li, Shan Muhammad, Diange Li, Zeyue Zhang, Yibo Gao, Qian Liu

**Affiliations:** 1 Department of Colorectal Surgery, National Cancer Center/National Clinical Research Center for Cancer/Cancer Hospital, Chinese Academy of Medical Sciences and Peking Union Medical College, Beijing, 100021 China; 2 Department of Operating Rooms, National Cancer Center/National Clinical Research Center for Cancer/Cancer Hospital, Chinese Academy of Medical Sciences and Peking Union Medical College, Beijing, 100021 China; 3 Laboratory for Advanced Medicine & Health Ltd. (LAMH), Beijing, 100176 China; 4 Department of Thoracic Surgery, National Cancer Center/National Clinical Research Center for Cancer/Cancer Hospital, Chinese Academy of Medical Sciences and Peking Union Medical College, Beijing, 100021 China; 5 Laboratory of Translational Medicine, National Cancer Center/National Clinical Research Center for Cancer/Cancer Hospital, Chinese Academy of Medical Sciences and Peking Union Medical College, Beijing, 100021 China; 6 State Key Laboratory of Molecular Oncology, National Cancer Center/National Clinical Research Center for Cancer/Cancer Hospital, Chinese Academy of Medical Sciences and Peking Union Medical College, Beijing, 100021 China; 7 Central Laboratory & Shenzhen Key Laboratory of Epigenetics and Precision Medicine for Cancers, National Cancer Center/National Clinical Research Center for Cancer/Cancer Hospital & Shenzhen Hospital, Chinese Academy of Medical Sciences and Peking Union Medical College, Shenzhen, 518116 China

**Keywords:** Cell-free DNA, Hypermethylation, Colorectal cancer, Prospective validation, Populational screening

## Abstract

**Background:**

Although colonoscopy is the standard screening test for colorectal cancer (CRC), its use is limited by a poor compliance rate, the need for extensive bowel preparation, and the risk of complications. As an alternative, an FDA-approved stool-based DNA test, Cologuard, has demonstrated satisfactory detection performance for CRC, but its compliance rate remains suboptimal, primarily attributable to individuals’ reluctance to provide stool samples.

**Methods:**

We developed a noninvasive blood-based CRC test, ColonSecure, based on cell-free DNA containing cancer-specific CpG island methylation patterns. We initially screened publicly available datasets for differentially methylated CpG sites in CRC with prediction potential. Subsequently, we performed two sequential bisulfite-free methylation sequencing on blood samples obtained from CRC patients and non-cancer controls. Through rigorous evaluation of each marker and machine learning-assisted feature selection, we identified 149 hypermethylated markers from over 193,000 CpG sites. These markers were then utilized to construct the ColonSecure model, enabling accurate CRC detection.

**Results:**

We validated the efficacy of our cell-free DNA methylation-based blood test for CRC screening with 3493 high-risk individuals identified from 114,136 urban residents. The ColonSecure test identified 89 out of 103 CRC patients diagnosed by the follow-up colonoscopy, outperforming CEA, CRP, and CA19-9 (with a sensitivity of 86.4% compared to 45.6%, 39.8%, and 25.2% for CEA, CRP, and CA19-9 respectively; an AUROC of 0.956 compared to an AUROC of < 0.77 for other methods).

**Conclusion:**

Our observations emphasize the potential of our multiple cfDNA methylation marker-based test for CRC screening in high-risk populations.

**Supplementary Information:**

The online version contains supplementary material available at 10.1186/s12943-023-01866-z.

## Introduction

Colorectal cancer (CRC) ranks as the third most prevalent cancer worldwide, displaying a consistently escalating incidence rate on a global scale, particularly in urban areas [1, 2]. Early detection and surveillance colonoscopic screening are effective in reducing CRC incidence and mortality [3]. Colonoscopy is the gold-standard visual test, but it is limited by suboptimal compliance rate due to invasiveness, the need for extensive bowel preparation, and the risk of complications. Other screening tests, such as fecal occult-blood testing (FOBT), fecal immunochemistry testing (FIT), sigmoidoscopy, and computed tomography (CT) colonography, are limited by poor sensitivity and/or specificity [4]. These challenges highlight the need for the development of simple, non-invasive, easy-to-detect, and cost-effective molecular screening tests that are specific for asymptomatic early-stage cancers.

Earlier studies have identified CRC specific DNA methylation sites including *SEPT9* [5–9], *VIM* [10], *BCAT1,* and *IKZF1* [11]. Despite being approved by the Food and Drug Administration (FDA) as a screening test for early stages, independent investigations have reported the sensitivity of *mSEPT9* assay to be between 39% and 88.9%, with specificity ranging between 79 and 100% [12]. Other candidate methylation biomarkers have been reported to exhibit similar sensitivity/specificity profiles in independent prospective studies [13]. By detecting methylation, mutation, and hemoglobin, Cologuard exhibits 92.3% sensitivity and 86.6% specificity in a prospective study of 9989 participants, but has a compromised compliance rate due to the inconvenience of collecting stool samples [14, 15].

In this study, we developed ColonSecure, a blood-based test, for CRC screening by using a panel of 149 methylation markers selected from two consecutive deep sequencing datasets generated in-house. We further evaluated the efficacy of this cfDNA methylation-based test in a populational prospective screening setting involving 114,136 urban residents.

## Materials and methods

### Participants enrollment

#### Retrospective patient enrollment

To build a CRC detection model (training set) and evaluate its performance (test set), we used retrospective blood samples of individuals diagnosed with CRC or at high-risk of developing CRC. The CRC patients were diagnosed by colonoscopy and histopathology examination according to current practice guidelines in China. The CRC stage was determined according to the 8^th^ American Joint Commission on Cancer (AJCC) TNM system. The high-risk subjects had at least one of the following conditions: (i) family history of CRC, (ii) colorectal adenoma or history of colorectal adenoma, (iii) inflammatory bowel diseases, and (iv) familial adenomatous polyposis. This retrospective cohort was collected from the Cancer Hospital Chinese Academy of Medical Sciences (Beijing, China) and the First Affiliate Hospital of Guangzhou Medical University (Guangzhou, China). The clinical characteristics and demographics of the patients are provided in Tables S1 and S2.

#### Prospective CRC screening cohort

For evaluating the model performance prospectively, we selected CRC high-risk participants from a multi-center, community-based population cohort which consists of 114,136 urban residents enrolled during 2020–2022 from Beijing, Hebei and Guangzhou. Urban residents were first requested to take an established clinical questionnaire to assess their risk of developing CRC. Only those individuals with informed consent signed, aged 20 to 90 years, and meeting one of the following criteria were considered as high-risk participants in this study: (i) family history of CRC, (ii) colorectal adenoma or history of colorectal adenoma, (iii) inflammatory bowel diseases, (iv) familial adenomatous polyposis, and (v) changes in bowel habits or stool appearance.

**Fig. 1:**
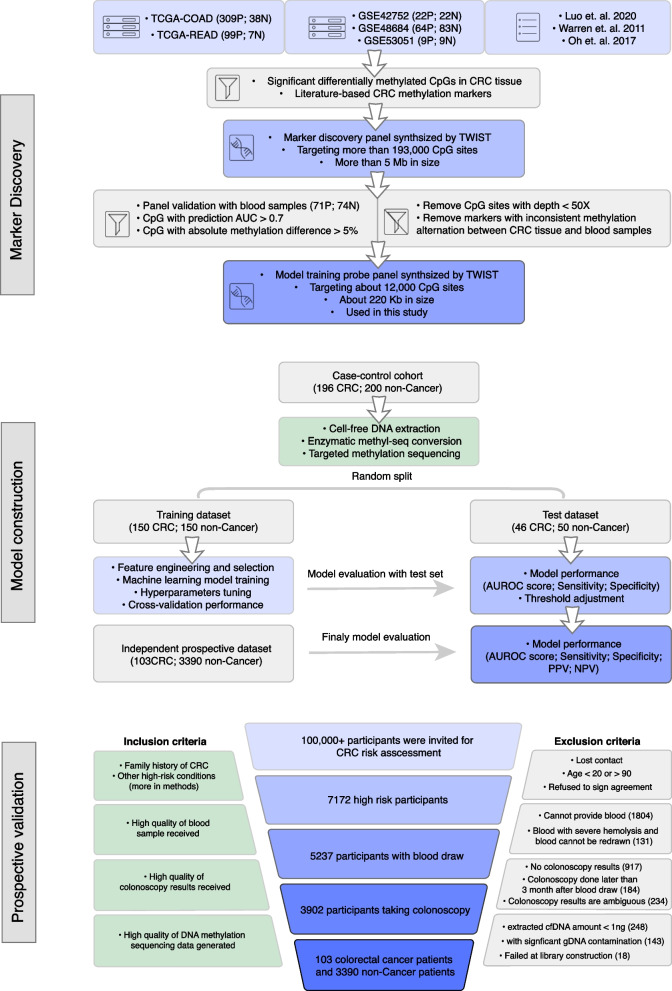
Workflow of three major phases for this study: marker discovery, model construction and prospective validation. During the marker discovery phase, two sequential targeted bisulfite sequencing experiments were conducted to identify potential methylation biomarkers with significant methylation alternation in both tissue and blood samples of CRC patients. The first marker discovery probe panel was synthesized based on methylation markers identified from TCGA/GEO databases and previous studies. The second model training probe panel was designed based on the markers further identified through analysis of clinical blood samples. P: positive group; N: negative group. The box icons, appearing in sequence, represent the databases, literature, selection/filtering criteria, and capture probe panel used. During the model construction phase, targeted methylation sequencing using the model training probe panel was performed on 396 clinical blood samples, which were split into training and test sets for model training and evaluation. To prospectively validate the methylation model, 3493 CRC high-risk participants were selected from urban residents based on inclusion and exclusion criteria

The high-risk participants were then consecutively enrolled if they (i) completed the questionnaire and signed the enrollment agreement, (ii) were able and willing to provide blood samples, and (iii) were able and willing to undergo colonoscopy within three months after blood draw. Finally, 3493 high-risk individuals with blood samples, colonoscopy and cfDNA methylation data that were compliant with quality control were included in the study (Fig. 1). The diagnosis of CRC was based on the colonoscopy results and follow-up pathological examination according to the clinical practice in China. The clinical characteristics and demographics of the enrolled high-risk participants are provided in Tables S3.

### Experiments and data process

#### Serum sample collection

Blood samples were collected using 5 mL BD Vacutainer® SST TM tubes and processed using routine protocols. Serum samples were transported to testing facility (Youze lab) on dry ice and aliquoted into barcoded cryovials for long-term storage at − 80 °C or below.

#### cfDNA extraction

Cell free DNA (cfDNA) was extracted from 2.5 mL serum using the Circulating DNA Extraction kit (GuangzhouYouze Biotech, China) according to manufacturer’s instructions. Briefly, protein was digested and removed from serum samples using Proteinase K. cfDNA was then bound to the spin column, purified, and eluted. Finally, cfDNA abundance was quantified by using the Qubit fluorometric method.

#### cfDNA library construction and sequencing

Next-generation sequencing (NGS) libraries were constructed with 5 ng cfDNA input using the NEBNext® Enzymatic Methyl-seq (EM-seq™) kit and Unique Dual Index Primer pairs (New England Biolabs, USA) according to the instructions from manufacturers. The EM-converted libraries were hybridized with a customized capture probe panel (Twist Bioscience, USA) to enrich for cfDNA molecules overlapping the regions of interest. After hybridization, the quality of final libraries was assessed using High-Sensitivity DNA chips on the Agilent Bioanalyzer. Finally, high-quality libraries were sequenced on the NovaSeq 6000 platform (Illumina, USA).

#### Methylation sequencing data analysis

Raw DNA methylation sequencing data was first analyzed by FastQC (v0.11.9) to examine the general sequencing quality. TrimGalore (v0.6.5) was then used to trim the contaminated adapter sequence, low-quality sequence (Phred score < 20), and sequence from 5’ end with severe M-bias. The trimmed FASTQ files were aligned to the hg19 human genome reference using BSMAP (v2.90) with default parameters. The aligned BAM files were processed by Picard (v2.22.7) to mark duplicates from PCR or optical sensor during sequencing. Samtools (v1.9) was used to remove unmapped reads, reads that are not primary alignments, and reads that are PCR or optical duplicates. The cleaned BAM files were further processed by alignmentSieve (Deeptools, v3.5.0) to filter out long fragments that are more than 200 bp which are potential contaminants of genomic DNA. Finally, the methratio.py script (BSMAP) was used to extract the methylation ratio for each CpG site covered by the targeted sequencing panel.

#### Model construction

Logistic regression (R package ‘glmnet’, v4.1.6), Random Forest (R package ‘ranger’, v0.14.1), Support Vector Machine (R package ‘e1071’, v1.7.12) and XGBoost (R package ‘xgboost’, v1.6.0.1) were used to construct cfDNA methylation-based prediction model with training set samples. Hyperparameter fine-tuning for each model was implemented by R package ‘caret’ (v6.0.93). The mean AUC score of 20 times repeated fivefold cross-validation was used for model comparison.

#### Sample size estimation for the prospective study

Based on the previous reports and the training sample set of this study, blood serum protein biomarkers demonstrated AUC scores ranging from 70 to 80% for CRC prediction. We hypothesized that the ColonSecure test could have a significantly elevated AUC score of 95%. To ascertain the necessary sample size, we used the ‘Two ROC Curves Power Analysis’ functionality provided by PASS 11.0 (UCSS, USA). With a two-sided significance level (alpha) at 0.05, 3200 high-risk participants will be required to secure a power of 80% to detect the enhanced AUC score of the methylation test in the prospective screening cohort.

#### Statistical analysis

Permutation-based Wilcoxon signed-rank test (10,000 permutations) was used to compare AUROCs between cfDNA methylation-based models and common protein biomarkers for CRC. The comparisons of sensitivity and specificity were performed by using McNemar’s test for paired proportions. A two-tailed p value less than 0.05 was considered as statistically significant. Prism software (v8.0) was used for statistical analyses. P values of the comparisons of AUROC and sensitivity between CRC methylation model and protein markers were provided in Tables S10 and S11, respectively.

## Results

### Marker discovery and probe panel design

To identify CpG sites that are differentially methylated in CRC, we collected the 450 K methylation array data of 662 samples from ‘The Cancer Genome Atlas Colon Adenocarcinoma (TCGA-COAD) and Rectum Adenocarcinoma (TCGA-READ) collection’, and GSE53051, GSE48684, and GSE42752 from the Gene Expression Omnibus (GEO) database. We selected differentially methylated CpG sites that exhibited an absolute methylation difference of ≥ 0.2 and an AUROC of ≥ 0.9. Based on the selected CRC-specific, differentially methylated CpG sites, we designed a targeted sequencing panel (Panel 1: marker discovery panel) targeting approximately 193,000 CpG sites with a size of approximately 5 MB.

Using this panel, we performed targeted sequencing using blood samples from 71 CRC patients and 74 controls (internal discovery group). CpG sites with coverage less than 50X and markers with inconsistent methylation alteration between CRC tissue and blood samples were excluded from downstream analysis. We then examined the performance characteristics of the targeted CpG sites in discriminating CRC from controls using univariate analysis. Based on AUROC (≥ 0.7) and absolute methylation difference (≥ 0.05), we then designed the second probe panel (Panel 2: model training panel) including approximately 12,000 CpG sites with a much smaller size of 220 kb, which enabled the generation of deeper sequencing data (Fig. 1).

### Methylation model training and evaluation

**Fig. 2:**
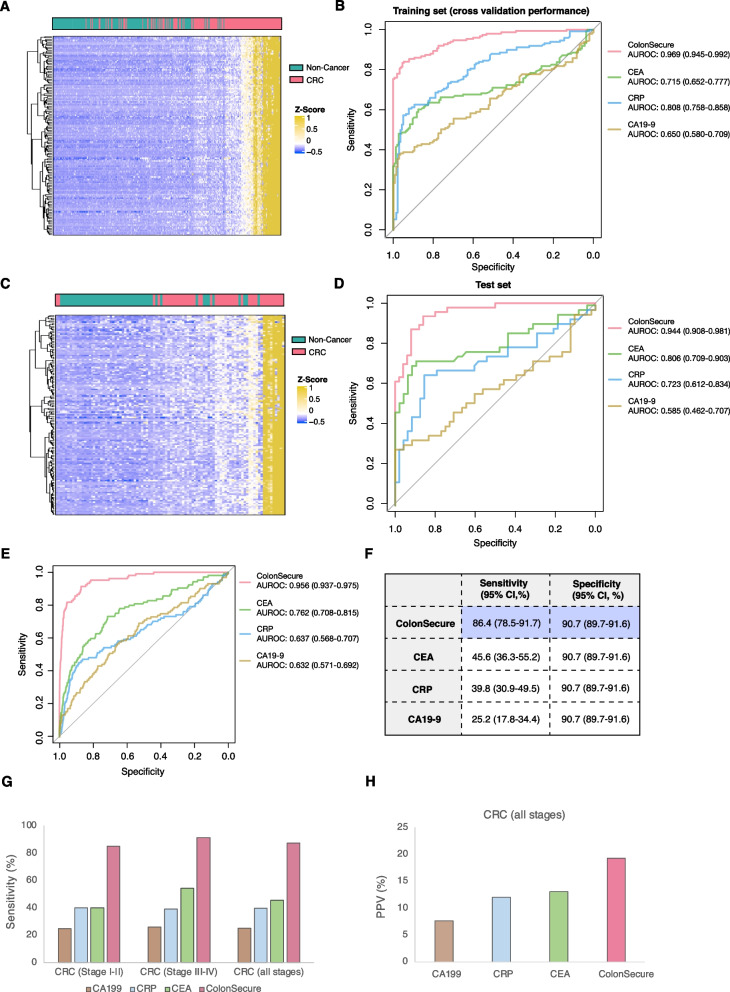
The construction of a cfDNA methylation-based CRC screening model and validation by a prospective high-risk population cohort. **A** Hierarchically clustered heatmap demonstrating the cfDNA methylation increment of the selected markers in CRC patients of training set. **B** ROC plots the of cfDNA methylation-based model and commonly used protein biomarkers for predicting CRC in training set. Random forest was used for training the cfDNA methylation-based model. The ROC curve of the methylation model was plotted based on out-of-bag prediction results. **C** Hierarchically clustered heatmap showing the methylation alternations of the selected markers in CRC patients of test set. **D** ROC curves of the methylation model and protein biomarkers for CRC detection in test set. **E** ROC curves for CRC detection by the methylation model and protein biomarkers in the prospective cohort. **F** Overall sensitivity and specificity. **G** Sensitivity of the CRC detection model for early and late-stage CRC. **H** Positive prediction values for cfDNA methylation-based model and protein markers

To generate a cfDNA methylation-based prediction model for CRC, we performed targeted deep sequencing using Panel 2 on blood samples collected from 196 CRC and 200 non-cancer subjects, which were randomly allocated to training and test groups (Fig. 1). By using the ‘Boruta’ feature selection package in the training dataset, we identified 149 informative hypermethylated markers. Unsupervised hierarchical cluster analysis revealed clear distinction between CRC and control samples based on the selected markers (Fig. 2A and C). By cross-validation in the training set, we determined the prediction model structure and fine-tuned the hyper-parameters to achieve optimal performance (Figure S1). To establish the utility of our methylation screening model, we compared it to conventional serum biomarkers that have been used for the detection of CRC – serum carcinoembryonic antigen (CEA), C-reactive protein (CRP) and serum carbohydrate antigen 19–9 (CA19-9). The thresholds for CRC prediction in the methylation model and protein biomarkers were established by maintaining a specificity of 90% within the test set. Our model exhibited superior sensitivity for discriminating CRC from controls in both the training and test datasets (Fig. 2B and D; Figure S2). The characteristics and ROC curves of the top 10 methylation markers are provided in Table S4 and Figure S3, respectively.

### Prospective validation

For evaluating the model performance prospectively, we enrolled CRC high-risk populations from 114,136 urban residents for CRC screening. Participants were first asked to take an established clinical questionnaire to assess their risk of developing CRC. High-risk participants were then invited to provide blood sample and take follow-up colonoscopy examination. Blood samples were collected and transported to the center lab for cfDNA extraction, methylation and protein biomarker profiling, and CRC prediction. These steps preceded the acquisition of colonoscopy diagnostic outcomes, which were independently provided by local physicians who were blinded to the methylation and protein test results. Only individuals with blood samples, colonoscopy, and cfDNA methylation data that met quality control standards were included in the study. Based on clinical cancer risk assessment questionnaires and inclusion/exclusion criteria, 3493 high-risk individuals were finally included for prospective validation of the ColonSecure test. Through colonoscopy and pathological examination, a total of 103 participants were diagnosed with colorectal cancer in three months after their blood was collected for the cfDNA methylation-based test (Fig. 1).

In this prospective CRC screening study, our cfDNA methylation-based ColonSecure test demonstrated significantly higher AUROC (0.956; 95% CI: 0.937–0.975) compared to CEA, CRP, and CA19-9 (Fig. 2E). By applying the cutoff determined at the model development phase, our test exhibited a sensitivity of 86.4% (95% CI: 78.5–91.7%) and a specificity of 90.7% (95% CI: 89.7–91.6%) in this prospective cohort while the protein biomarkers only exhibited suboptimal sensitivities (Fig. 2F). More importantly, our test predicted 34 out of 40 participants with early-stage (I and II) CRC (sensitivity of 85.0%; 95% CI 70.9–92.9%) which was superior to protein biomarkers (Fig. 2G and Tables S5–S11).

To avoid the overdiagnosis of benign colorectal lesions which require no interventions, patients with non-cancer colorectal diseases, such as adenoma, were included in the negative control group during the construction of the ColonSecure model. Among the 1735 high-risk participants who were diagnosed with benign adenoma, our model predicted 1556 as negative with a specificity of 89.7% (95% CI: 88.2–91.0%), which was comparable to the specificity of overall non-cancer participants (90.7%; 95% CI: 89.7–91.6%) (Table S5). The ColonSecure test also exhibited a significantly higher positive predictive value (19.3%; 95% CI: 16.0–23.1%) in predicting all stages of CRC compared to CEA, CRP, and CA19-9 (Fig. 2H).

## Discussion

Despite the effectiveness of colonoscopy as a screening test for CRC, its invasiveness, suboptimal compliance rate, need for thorough bowel preparation, and inherent inter-observer variability pose significant limitations to its widespread utilization. In contrast, liquid biopsy offers a non-invasive approach for screening and monitoring CRC through the detection of tumor-derived DNA circulating in the bloodstream, which carries specific genetic and epigenetic alterations associated with cancer. This technique provides a promising avenue for non-invasive assessment and surveillance of CRC, overcoming several limitations associated with traditional screening methods. In this study, we developed a CRC screening model based on 149 cfDNA methylation markers and assessed the potential of this model in effectively discriminating CRC patients from control subjects. The cfDNA methylation-based ColonSecure test exhibited exceptional sensitivity (85.3% and 87.0% respectively) at 90% specificity in discriminating CRC patients from non-cancer controls in both training and test groups, outperforming conventional blood tests – CEA, CRP and CA19-9. The robust detection performance of our test was subjected to further validation in a prospective study involving a high-risk population. Compared to the *mSEPT9* blood test investigated at similar settings, our ColonSecure test demonstrated significantly increased sensitivity and comparable specificity [12]. While having comparable detection performance with the stool-based Cologuard test, which has demonstrated superiority over the widely-used FIT test, our blood-based test is anticipated to have a significantly improved compliance rate which is crucial for the application of cancer screening [14, 15]. This indicates that the ColonSecure test is potentially optimal for CRC screening in high-risk populations.

Due to the COVID-19 pandemic, a portion of the initially enrolled high-risk participants were unable to fulfill their appointments for blood collection or colonoscopy examination, leading to their exclusion from the study and consequently yielding an underestimated compliance rate. An additional 266 high-risk subjects were excluded from the study due to exceedingly low cfDNA amount or library construction failure. The implementation of automated instruments for stabilized sample processing holds the potential to alleviate these issues. Another notable limitation inherent in our study design was the absence of follow-up for participants who exhibited a positive prediction for CRC based on the ColonSecure test but were found to be CRC-free upon colonoscopy examination. The utilization of serum samples, which exhibit higher genomic DNA background noise, for cfDNA methylation profiling is also suboptimal. The inclusion of follow-up data and plasma-based validation in future investigations holds promise for a more comprehensive evaluation of the test’s performance. Additionally, age was shown to be a confounding factor to the ColonSecure methylation model, suggesting that its performance could exhibit variability when applied to cohorts characterized by diverse age distributions. Finally, the validation of the clinical utility of the test warrants additional large-scale randomized controlled studies.

### Supplementary Information

**Additional file 1: Supplementary Figure S1.** Comparison of machine learning models for CRC detection by cross validation in training set. **Supplementary Figure S2.** Developing cfDNA methylation-based model for CRC detection. **Supplementary Figure S3.** ROC plot for top 10 methylation markers used for the methylation model construction. **Table S1.** Clinical characteristics of the case-control samples for marker discovery. **Table S2.** Clinical characteristics of the case-control samples for model construction. **Table S3.** Clinical characteristics of the prospective cohort for CRC screening. **Table S4.** Characteristics of the top 10 methylation markers used in the CRC prediction model. **Table S5.** Prediction performance of the methylation model in prospective CRC high-risk cohort. **Table S6.** Prediction performance of CEA in prospective CRC high-risk cohort. **Table S7.** Prediction performance of CRP in prospective CRC high-risk cohort. **Table S8.** Prediction performance of CA19-9 in prospective CRC high-risk cohort. **Table S9.** Confounding factor analysis for the ColonSecure test. **Table S10.** Permutation-based Wilcoxon signed-rank test P values for comparison of test AUROC. **Table S11.** McNemar’s test *P* values for comparison of test sensitivity.

## Data Availability

Sequencing data generated in this study are available upon request.

## Abbreviations

cfDNA: Cell-free DNA
CRC: Colorectal cancer
FOBT: Occult-blood testing
FIT: Fecal immunochemistry test
CT: Computed tomography
AUROC: Area under the receiver operating characteristic curve
CEA: Carcinoembryonic antigen
CRP: C-Reactive Protein
CA19-9: Carbohydrate antigen 19–9
