# Supplementary material for: Efficacy of cell-free DNA methylation-based blood test for colorectal cancer screening in high-risk population: a prospective cohort study
Source: Mol Cancer. 2023 Sep 28;22:157. doi: 10.1186/s12943-023-01866-z (PMC10538018; doi:10.1186/s12943-023-01866-z)
Supplement: Supplementary file 1 — Additional file 1: Supplementary Figure S1. Comparison of machine learning models for CRC detection by cross validation in training set. Supplementary Figure S2. Developing cfDNA methylation-based model for CRC detection. Supplementary Figure S3. ROC plot for top 10 methylation markers used for the methylation model construction. Table S1. Clinical characteristics of the case-control samples for marker discovery. Table S2. Clinical characteristics of the case-control samples for model construction. Table S3. Clinical characteristics of the prospective cohort for CRC screening. Table S4. Characteristics of the top 10 methylation markers used in the CRC prediction model. Table S5. Prediction performance of the methylation model in prospective CRC high-risk cohort. Table S6. Prediction performance of CEA in prospective CRC high-risk cohort. Table S7. Prediction performance of CRP in prospective CRC high-risk cohort. Table S8. Prediction performance of CA19-9 in prospective CRC high-risk cohort. Table S9. Confounding factor analysis for the ColonSecure test. Table S10. Permutation-based Wilcoxon signed-rank test P values for comparison of test AUROC. Table S11. McNemar’s test P values for comparison of test sensitivity. [file 12943_2023_1866_MOESM1_ESM.pdf]

Supplementary Materials for

Efficacy of cell-free DNA methylation-based blood test for colorectal cancer screening in high-risk population: a prospective cohort study

Fuqiang Zhao[1,8], Ping Bai[2,8], Jianfeng Xu[3,8], Zitong Li[4,8], Shan Muhammad[4], Diange Li[3], Zeyue Zhang[3], Yibo Gao[4-7*], Qian Liu[1*]

Correspondence to: gaoyibo@cicams.ac.cn (Y. G.) and liuqian@cicams.ac.cn (Q. L.)

This PDF file includes:

Supplementary Figs. S1 to S3

Supplementary table S1 to S11

Supplementary Figures

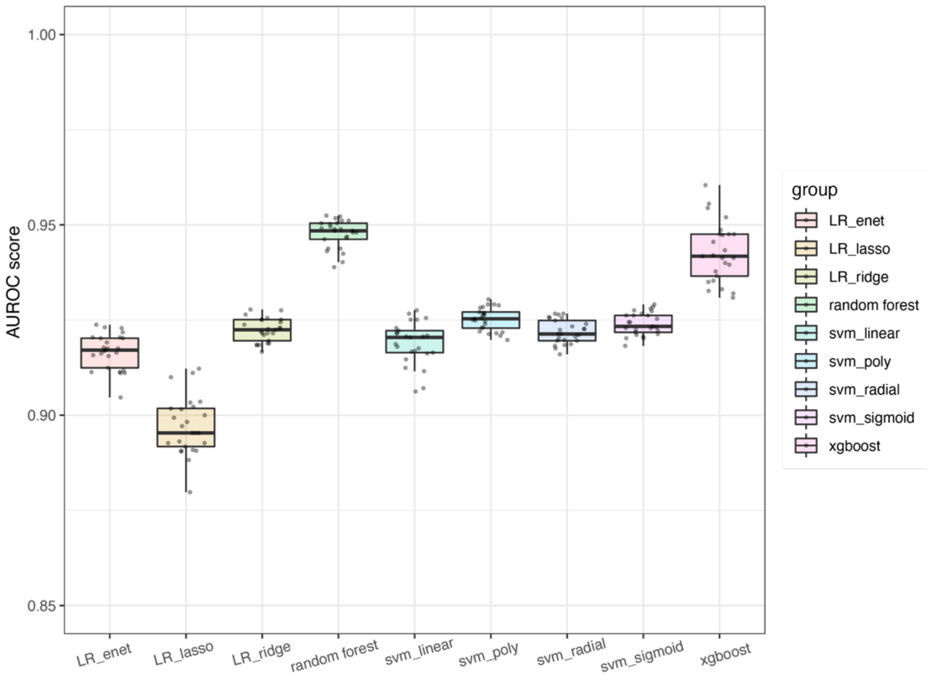

Supplementary Figure S1. Comparison of machine learning models for CRC detection by cross-validation in training set.

A

Training set	Sensitivity (95% CI, %)	Specificity (95% CI, %)
**ColonSecure**	85.3 (78.8-90.1)	90.0 (84.2-93.9)
**CEA**	49.3 (41.4-57.2)	90.0 (84.2-93.9)
**CRP**	57.3 (49.3-64.9)	90.0 (84.2-93.9)
**CA19-9**	39.3 (31.8-47.3)	90.0 (84.2-93.9)

B

Test set	Sensitivity (95% CI, %)	Specificity (95% CI, %)
**ColonSecure**	87.0 (74.4-93.9)	90.0 (78.6-95.7)
**CEA**	58.7 (44.3-71.7)	90.0 (78.6-95.7)
**CRP**	41.3 (28.3-55.7)	90.0 (78.6-95.7)
**CA19-9**	34.8 (22.7-49.3)	90.0 (78.6-95.7)

Supplementary Figure S2. Developing cfDNA methylation-based model for CRC detection. The sensitivity and specificity of the methylation model and protein biomarkers for training set (A) and test set (B).

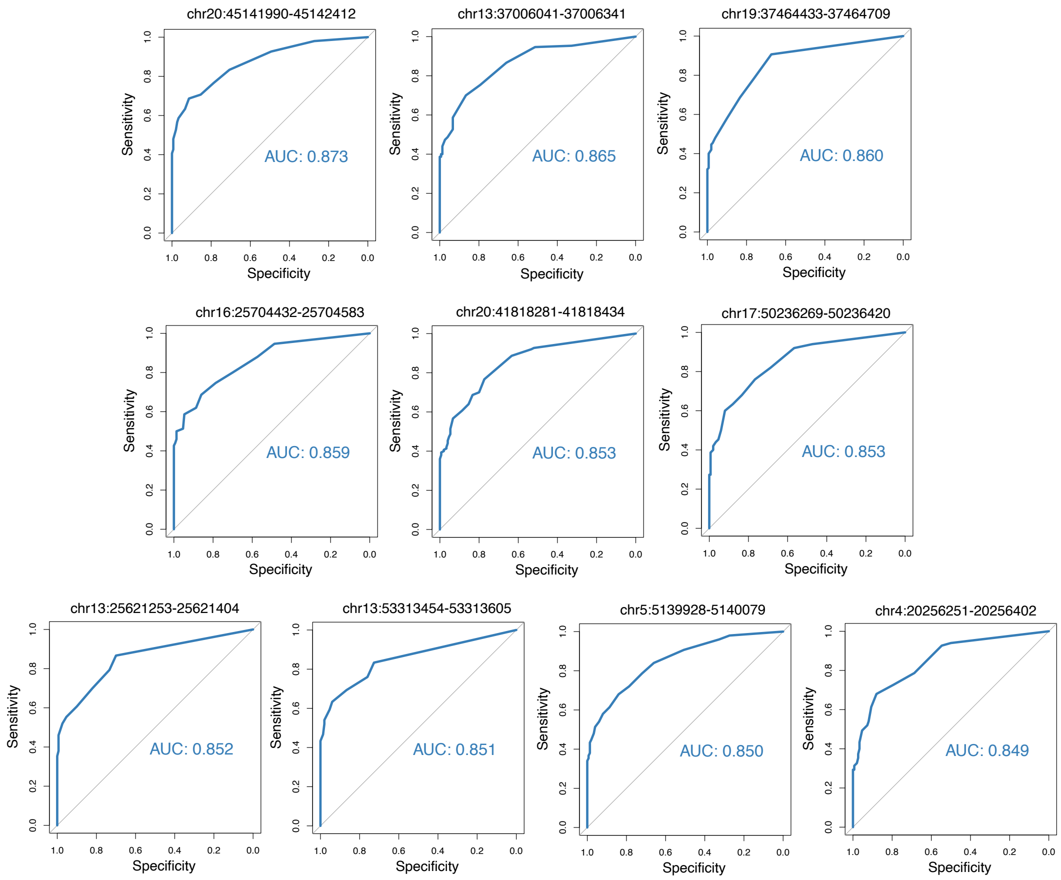

Supplementary Figure S3. ROC plot for top 10 methylation markers used for the methylation model construction.

Supplementary Tables

Table S1 Clinical characteristics of the case-control samples for marker discovery

	CRC	Non-cancer subjects
**Subjects (n)**	71	74
**Age, median, years**	66	59
**Sex**		
Male (n)	41 (57.7%)	31 (41.9%)
Female (n)	30 (42.3%)	43 (58.1%)
**Stage**		
I	9 (12.7%)	
II	11 (15.5%)	
III	25 (35.2%)	
IV	26 (36.6%)	
**Non-cancer conditions**		
Family history of CRC	NA	21 (28.4%)
Adenoma or history of adenoma	NA	39 (52.7%)
Inflammatory bowel disease	NA	20 (27.0%)
Familial adenomatous polyposis	NA	3 (4.1%)

Table S2 Clinical characteristics of the case-control samples for model construction

	CRC	Non-cancer subjects
**Subjects (n)**	196	200
**Age, median, years**	61	60
**Sex**		
Male (n)	120 (61.2%)	121 (60.5%)
Female (n)	76 (38.8%)	79 (39.5%)
**Stage**		
0	4 (2.0%)	
I	12 (6.1%)	
II	18 (9.2%)	
III	52 (26.5%)	
IV	42 (21.4%)	
Unstaged or unknown	68 (34.7%)	
**Non-cancer conditions**		
Family history of CRC	NA	40 (20.0%)
Adenoma or history of adenoma	NA	146 (73.0%)
Inflammatory bowel disease	NA	43 (21.5%)
Familial adenomatous polyposis	NA	5 (2.5%)

Table S3 Clinical characteristics of the prospective cohort for CRC screening

	CRC	Non-cancer subjects
**Subjects (n)**	103	3390
**Age, median, years**	62	54
**Sex**		
Male (n)	57 (55.3%)	1723 (50.8%)
Female (n)	46 (44.7%)	1667 (49.2%)
**Stage**		
0	3 (3.0%)	
I	12 (11.7%)	
II	25 (24.3%)	
III	36 (35.0%)	
IV	10 (9.7%)	
Unstaged or unknown	17 (16.5%)	
**Non-cancer conditions**		
Family history of CRC	NA	281 (8.3%)
Adenoma or history of adenoma	NA	2259 (66.7%)
Inflammatory bowel disease	NA	531 (15.7%)
Familial adenomatous polyposis	NA	13 (0.4%)

Table S4 Characteristics of the top 10 methylation markers used in the CRC prediction model

Marker ID	Chr	Start	End	Gene Name	Annotation	Distance to TSS	AUC	Sensitivity at 90% specificity
**CRC_1**	chr20	45141990	45142412	ZNF334	promoter	-3	0.873	68.7%
**CRC_2**	chr13	37006041	37006341	CCNA1	promoter	-218	0.865	58.7%
**CRC_3**	chr19	37464433	37464709	ZNF568	intron	57340	0.860	58.0%
**CRC_4**	chr16	25704432	25704583	HS3ST4	intron	1161	0.859	58.7%
**CRC_5**	chr20	41818281	41818434	PTPRT	exon	199	0.853	56.7%
**CRC_6**	chr17	50236269	50236420	CA10	promoter	-213	0.853	60.0%
**CRC_7**	chr13	25621253	25621404	PABPC3	Intergenic	-48947	0.852	60.7%
**CRC_8**	chr13	53313454	53313605	LECT1	intron	417	0.851	63.3%
**CRC_9**	chr5	5139928	5140079	CTD-2297D10.2	promoter	163	0.850	58.0%
**CRC_10**	chr4	20256251	20256402	SLIT2	intron	1140	0.849	61.3%

Table S5 Prediction performance of the methylation model in prospective CRC high-risk cohort

Methylation model	Participants	Predicted Positive	Predicted Negative	Sensitivity (95% CI,%)	Specificity (95% CI, %)
CRC (Stage I-II)	40	34	6	85.0 (70.9-92.9)	
CRC (Stage III-IV)	46	42	4	91.3 (79.7-96.6)	
CRC (all Stages)	103	90	13	86.4 (78.5-91.7)	
Colon adenoma	1735	179	1556		89.7 (88.2-91.0)
All non-Cancer	3390	376	3014		90.7 (89.7-91.6)

Table S6 Prediction performance of CEA in prospective CRC high-risk cohort

CEA	Participants	Predicted Positive	Predicted Negative	Sensitivity (95% CI,%)	Specificity (95% CI, %)
CRC (Stage I-II)	40	16	24	40.0 (26.4-55.4)	
CRC (Stage III-IV)	46	25	21	54.3 (40.1-67.8)	
CRC (all Stages)	103	47	56	45.6 (36.3-55.2)	
Colon adenoma	1735	173	1562		90.0 (88.5-91.3)
All non-Cancer	3390	315	3075		90.7 (89.7-91.6)

Table S7 Prediction performance of CRP in prospective CRC high-risk cohort

CRP	Participants	Predicted Positive	Predicted Negative	Sensitivity (95% CI,%)	Specificity (95% CI, %)
CRC (Stage I-II)	40	16	24	40.0 (26.4-55.4)	
CRC (Stage III-IV)	46	18	28	39.1 (26.4-53.5)	
CRC (all Stages)	103	43	60	39.8 (30.9-49.5)	
Colon adenoma	1735	166	1569		90.4 (88.9-91.7)
All non-Cancer	3390	315	3075		90.7 (89.7-91.6)

Table S8 Prediction performance of CA19-9 in prospective CRC high-risk cohort

CA19-9	Participants	Predicted Positive	Predicted Negative	Sensitivity (95% CI,%)	Specificity (95% CI, %)
CRC (Stage I-II)	40	10	30	25.0 (14.2-40.2)	
CRC (Stage III-IV)	46	12	34	26.1 (15.6-40.3)	
CRC (all Stages)	103	26	77	25.2 (17.8-34.4)	
Colon adenoma	1735	175	1560		90.0 (88.5-91.3)
All non-Cancer	3390	314	3076		90.7 (89.7-91.6)

Table S9 Confounding factor analysis for the ColonSecure test

	Coefficient	Standard Error	z	P > |z|	Odds Ratio (OR)	OR Lower CI	OR Upper CI
**ColonSecure Result**	12.484	0.912	13.685	1.255E-42	2.640E5	4.826E4	1.737E6
**Intercept**	-13.987	1.050	-13.323	1.711E-40	8.426E-7	9.549E-8	5.901E-6
**Patient Demographics**							
Age	0.035	0.012	2.831	4.640E-3	1.035	1.011	1.061
Sex (male)	0.189	0.253	0.750	0.453	1.209	0.737	1.990
**Tumor site: R vs L**	-0.392	0.274	-1.432	0.152	0.676	0.391	1.146

Table S10 Permutation-based Wilcoxon signed-rank test P values for comparison of test AUROC

	CEA	CRP	CA19-9
**Training set**			
ColonSecure	< 0.001	< 0.001	< 0.001
**Test set**			
ColonSecure	< 0.001	< 0.001	< 0.001
**Prospective validation set**			
ColonSecure	< 0.001	< 0.001	< 0.001

Table S11 McNemar's test P values for comparison of test sensitivity

	CEA	CRP	CA19-9
**Training set**			
ColonSecure	4.5E-08	8.1E-13	4.2E-14
**Test set**			
ColonSecure	0.012	6.3E-05	1.4E-05
**Prospective validation set**			
ColonSecure	6.7E-09	3.3E-10	4.0E-13
**Prospective validation set (early stage)**			
ColonSecure	2.9E-04	1.8E-04	6.5E-06
